# Microfluidic Separation of a Soluble Substance Using Transverse Diffusion in a Layered Flow

**DOI:** 10.3390/mi8010009

**Published:** 2016-12-29

**Authors:** Xuan Don Nguyen, Hyeong Jin Jeon, Hyo Yong Kim, Hyun Jong Paik, June Huh, Hyung Hoon Kim, Jeung Sang Go

**Affiliations:** 1 School of Mechanical Engineering, Pusan National University, 2, Busandaehak-ro 63beon-gil, Geumjeong-gu, Busan 46241, Korea; nguyenxuandon@pusan.ac.kr (X.D.N.); hjjeon@pusan.ac.kr (H.J.J.); 2 Department of Polymer Science & Engineering, Pusan National University, 2, Busandaehak-ro 63beon-gil, Geumjeong-gu, Busan 46241, Korea; hyong0901@gmail.com (H.Y.K.); hpaik@pusan.ac.kr (H.J.P.); 3 Department of Chemical & Biological Engineering, College of Engineering, Korea Univeristy, Anam-Dong, Seongbuk-Gu, Seoul 02841, Korea; junehuh@korea.ac.kr; 4 Boditech Med Inc., 43, Geodudanji 1-gil, Dongnae-myeon, Chuncheon-si, Gang-won-do 24398, Korea; khh@boditech.co.kr

**Keywords:** transverse diffusion, layered flow, polymers, separation, microfluidic

## Abstract

This paper presents a practical flow-through method to separate anisole and ethyl phenylacetate, respectively, from a polystyrene mixture. The microfluidic separation uses different diffusive dynamics of the substances transverse to the lamination flow formed in a microchannel. The effect of inlet flow rates and ambient temperature on separation is examined. Additionally, the possibility of the separation of the light substance from the mixture with different molecular weight is shown numerically and experimentally. The separation efficiency is explained by the facts that the relaxation time depends on the inlet flow rate and that the diffusivity depends on the ambient temperature. This method can be applied to separate monomers from aggregates.

## 1. Introduction

The selective separation of a specific substance from a mixture is an important process in chemical, biochemical, and medical analysis. Various separation techniques such as filtration [1], capillary electrophoresis [2], liquid chromatography and mass spectrometry [3], bead handling [4], and ultracentrifugation [5] have been developed. However, very few works on the continuous separation of specific substances from mixtures dissolved in a solution have been reported. For example, the chromatography operates discontinuously and generally needs complex separation columns.

For the continuous separation, the microfluidic methods have been challenged to separate particles [6,7], cells [8,9], deoxyribonucleic acid (DNA) [10,11], or proteins [12] in recent years owing to their high potential of commercialization. A strong Dean flow in a curved microchannel [13], the pinched flow fractionation in a non-uniform flow [14], the deterministic lateral displacement in a pillar array [15], and microfiltration [16] have been introduced representatively. However, these methods always need an external field to apply for separation, and the size of particle has been limited. In this work, we report for the first time a continuous method for selectively separating a molecular species from a mixture, which relies on different diffusion characteristics of mixture components under a laminar flow in a microfluidic channel.

## 2. Materials and Methods

### 2.1. Numerical Analysis of Diffusion

**Figure 1:**
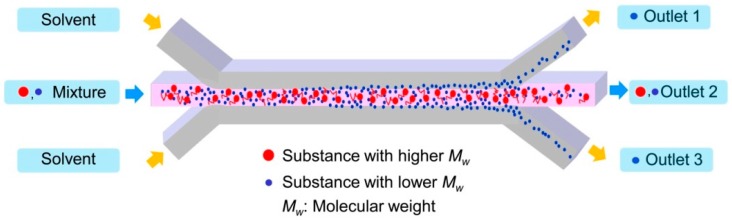
Separation of only small molecules from the mixture of two different molecules using the different transverse diffusion in a lamination flow.

Figure 1 describes the separation mechanism of the substance from the mixture of the molecules with different molecular weights. When the two molecules dissolved in the solvent are introduced through the central inlet, they meet the solvent flow injected from the side inlets. A laminar flow forms instantaneously at the junction and the stable interface between the solution containing the molecules and the solvent is generated. Through the interface of two laminated solutions, diffusion occurs due to the concentration gradient. Then, the small molecules diffuse through the interface faster than large molecules do. Consequently, only the small molecules can be collected at the side outlets by controlling the inlet flow rates. This flow-through separation of the substance from the mixture can be obtained simply by using the dynamic behavior of molecules with different diffusivities in the transverse direction to the channel flow.

As an attempt for this concept, the mixture of a high molecular weight substance and a low molecular weight substance was prepared to separate the low molecular weight substance from the mixture. Mixture 1 and 2 consist of polystyrene (PS) with an average molecular weight, *M*_n_, of 46,000 g/mol as the high molecular weight substance and two low molecular weight substances—anisole with an *M*_n_ of 108 g/mol and ethyl phenylacetate (EPA) with an *M*_n_ of 164 g/mol—were examined to separate anisole and EPA. These materials have been popularly used in chemistry and polymer science.

The microfluidic separation channel was designed to have 3 inlets to form a stable lamination flow at the junction and 3 outlets to collect anisole and EPA selectively from two side outlets. The length from the junction of the inlets to the by-pass junction of the outlets was 35 mm and the channel height and width were 100 µm and 500 µm, respectively.

The Zimm model [17] describes the polymer dynamics in a dilute solution. The diffusivity (*D*) and the relaxation time (τ) in the solvent are given as

(1) $$D\sim \frac{k_{B}T}{\eta_{s}M^{\nu }},\;\tau \sim \frac{\eta_{s}M^{3\nu }}{k_{B}T}$$

where *k*_B_ is the Boltzmann’s constant, *T* is the temperature, η_s_ is the viscosity of the solvent, *M* is the molecular weight of the solute, and the exponent ν is given by 0.588 in the solvent.

In the solution, the PS diffuses more slowly than the anisole and EPA owing to its large molecular weight. Additionally, the molecular weight of the molecule relates proportionally to the hydrodynamic radius [18]. The relaxation time of PS is roughly 4.3 × 10^4^ and 2.1 × 10^4^ times longer than that of anisole and EPA, respectively. The large difference of relaxation time between PS and anisole or EPA can be a sufficient dynamic contrast to separate anisole or EPA from the mixture using transverse diffusion.

To examine the possibility of the flow-through separation of the anisole or EPA from the mixture in the lamination flow using a different transverse diffusion, the numerical analysis of the diffusion was conducted for the PS and anisole. The inlet flow rate of *N*,*N*-dimethylformamide (DMF) containing the PS and anisole was 500 µL/min, and the pure DMF solvent of 1500 µL/min was introduced from the side inlets. To calculate the transverse diffusion of the molecules, the diffusion coefficients of anisole and PS were 10^−9^ m^2^/s and 10^−11^ m^2^/s, respectively, as inlet conditions [19]. The diffusion coefficient of PS is smaller than anisole because the molecular weight of PS is larger than that of anisole.

Diffusion coefficient can be influenced by the number of molecules, the molecular weight, and the hydrodynamic radius. In general, the increase in the number of molecules decreases the diffusion coefficient of solutes. Additionally, the high molecular weight and large hydrodynamic radius decrease the diffusion coefficient. Thus, the interfacial diffusion constant changes when the small molecules are separated from the mixture with large molecules. However, the software used in the simulation was limited to reflect the instantaneous change of the diffusion coefficient. Thus, the diffusion coefficient was varied as an input condition for the different molecules.

**Figure 2:**
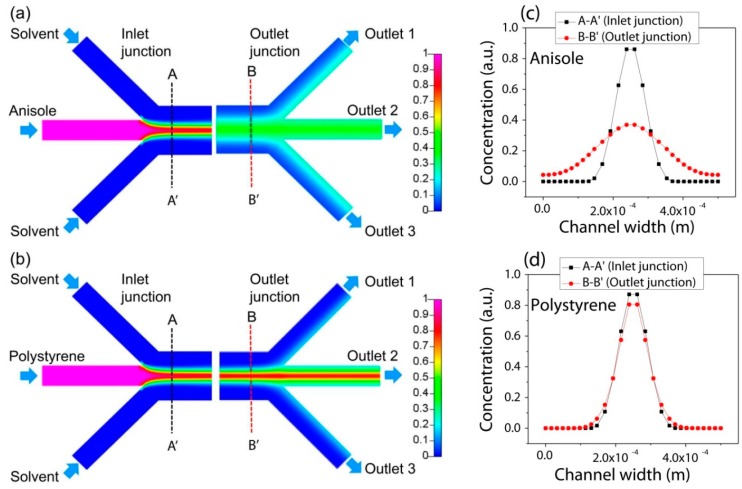
Numerical analysis of the concentration change along the channel length for an inlet flow rate of 500 µL/min of the mixture and 1500 µL/min of the solvent: (**a**) anisole; (**b**) polystyrene (PS); (**c**) concentration profile of anisole; (**d**) concentration profile of PS.

Figure 2 visualizes the concentration distribution along the channel. Specifically, the concentration profiles of PS and anisole along the line of A-A′ near the inlet junction and the line of B-B′ near the outlet junction compares their diffusion behavior between the inlets and outlets. The concentration of the anisole was distributed more broadly by diffusion through the interface of the lamination flow as shown in Figure 2a,c, while that of PS was kept almost the same as shown in Figure 2b,d in the transverse direction to the channel flow.

Thus, by using their different transverse diffusion, anisole can be only separated from the mixture of PS and anisole. Their mixture is introduced into the center channel of the three inlets, and it is focused by the side flow of the pure solvent. Then, PS and anisole in the solution can be positioned in the center of the microfluidic separation channel. Then, the flow-through anisole with a high diffusion coefficient diffuses fast compared with PS in the transverse direction. By manipulating the inlet flow rate, the transverse diffusion distance can be also controlled for the constant channel length. Finally, only anisole can be separated and collected from the side outlets.

### 2.2. Fabrication Process of the Flow-Through Separation Channel

**Figure 3:**
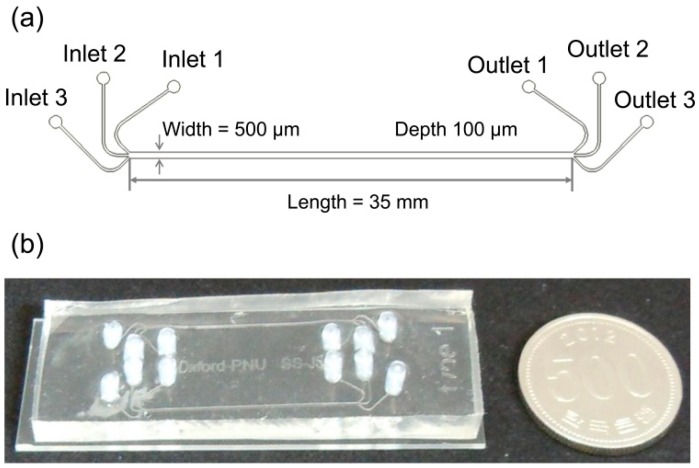
Design and fabrication of the microfluidic channel: (**a**) detailed sizes of the microfluidic channel; (**b**) picture of the fabricated microfluidic channel.

For the fabrication process, the microfluidic separation channel was fabricated by bonding the patterned polydimethylsiloxane (PDMS) and a transparent slide glass after treating oxygen plasma. Firstly, a 100-µm-thick layer of SU-8 photoresist (SU-8 2050, MicroChem Corp., Westborough, MA, USA) was spin-coated on a smooth silicon wafer, and photolithography was conducted to pattern the microfluidic separation channel. Then, the PDMS was poured onto the patterned SU-8 mold and cured at 65 °C for 2 h. The PDMS was detached from the SU-8 mold to form the pattern of the microfluidic separation channel on the PDMS. Finally, the patterned PDMS layer was bonded with the glass substrate after treating the surface with oxygen plasma (Plasma cleaner PDC-32G, Harrick Plasma Inc., Ithaca, NY, USA) for 2 min. The detailed dimensions and the picture of the fabricated microfluidic separation channel with the three inlets and three outlets were shown in Figure 3.

### 2.3. Experiment of Separation

The mixture of PS and anisole was prepared by dissolving PS of 33.34 mg and anisole of 0.166 mL in DMF of 2 mL. DMF was also introduced additionally through the two side inlets positioned symmetrically with respect to the center inlet into which the mixture solution was injected. The inlet flow rate of the mixture solution was regulated from 5 µL/min to 50 µL/min and that of the DMF solvent from 15 µL/min to 150 µL/min, respectively. The ratio of the inlet flow rates was kept 3.0. The separated solutions were collected through the two side outlets.

Similarly, we performed the experiment of the mixture of PS and EPA. To prepare this mixture, PS of 20 mg and EPA of 0.1 mL were dissolved in DMF of 2 mL. The experiment was then carried out by varying inlet flow rates of the mixture solution and the DMF solvent in the same process with the mixture of PS and anisole above. The intensity of the molecule was measured using gel permeation chromatography (GPC).

In the experiments, the molecules of anisole and PS were not balanced by considering the accuracy of the GPC measurement. To make a balance of the molecules between anisole and PS in the DMF solvent, the quantity of anisole in the mixture with PS should decrease from 0.166 mL to 7.87 × 10^−5^ mL, equivalent to 7.83 × 10^−5^ g. This amount of anisole makes the concentration of anisole in DMF solvent too low. It is not sufficient to measure the molecular weight using GPC analysis that requires at least 3 mg/mL to obtain clear peaks. Secondly, to make the balance of the number of molecules, the quantity of PS in the mixture is needed to increase. The quantity of PS should increase from 33.34 mg to 70.35 g. This quantity of PS results in a very high concentration in the DMF solvent, which is not recommended when measuring with GPC.

## 3. Results and Discussion

**Figure 4:**
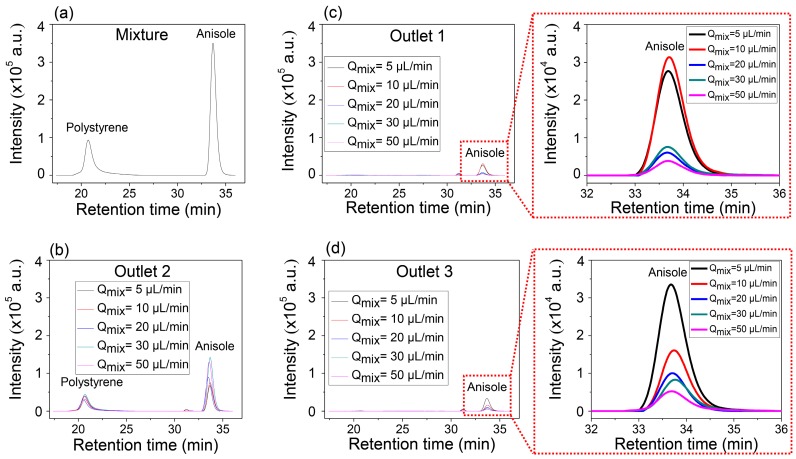
Selective continuous separation of anisole from the mixture of anisole with PS using gel permeation chromatography (GPC): (**a**) GPC analysis of the mixture; (**b**) GPC analysis of Outlet 2; (**c**) GPC analysis of Outlet 1; and (**d**) GPC analysis of Outlet 3 for the different inlet flow rates. *Q*_mix_: The inlet flow rate of the mixture.

Figure 4a,b show the GPC results of the mixture solution before injection in Figure 4a and that of the mixture collected in the center outlet (Figure 4b), respectively. Two separate peaks in Figure 4b indicate that the collected solution through Outlet 2 contains both anisole and PS. However, it is identical to the solutions collected from the two side outlets, Outlet 1 and Outlet 3, which show only single peak corresponding to the anisole (Figure 4c,d). It is also worth noting that the intensity of anisole increased in the side outlets as the inlet flow rate decreased, but the peak of PS was not detected. Anisole has enough time to transversely diffuse, while PS does not have enough time to diffuse so that it can be collected through the side outlets at the controlled conditions of the inlet flow rates and the constant length of the microfluidic separation channel.

**Figure 5:**
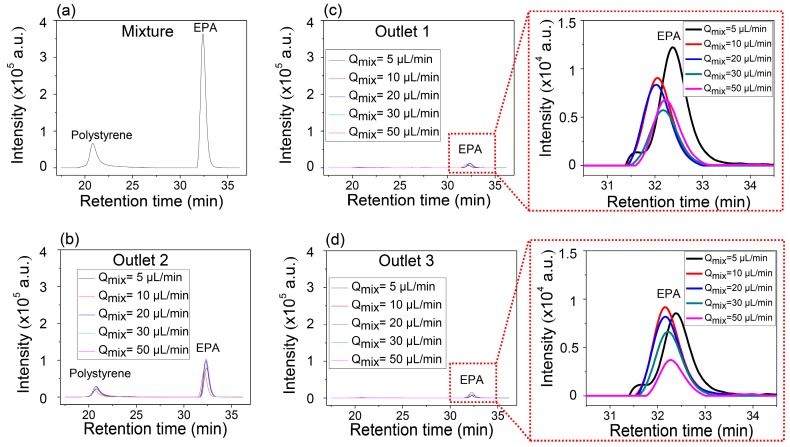
Selective continuous separation of ethyl phenylacetate (EPA) from the mixture of EPA with PS using GPC: (**a**) GPC analysis of the mixture; (**b**) GPC analysis of Outlet 2; (**c**) GPC analysis of Outlet 1; (**d**) GPC analysis of Outlet 3 for the different inlet flow rates.

The explanation of the separation possibility of EPA from the mixture with PS is similar to that of the mixture of PS and anisole. Even though the molecular weight of EPA is 1.5 times higher than that of anisole, the results also show the possibility of the separation of EPA from the mixture with PS dissolved in DMF solvent. However, the higher molecular weight of EPA has a smaller diffusion coefficient than anisole, and this means slower transverse diffusion, which results in the low intensity of EPA, separated from the mixture with PS, compared with the anisole separation. This can be well explained through the comparison of the GPC intensities at the magnified Figure 4c,d and Figure 5c,d. The highest value of the intensity of anisole at the side outlets was approximately 3 × 10^4^, while that of EPA was about 1.2 × 10^4^.

**Figure 6:**
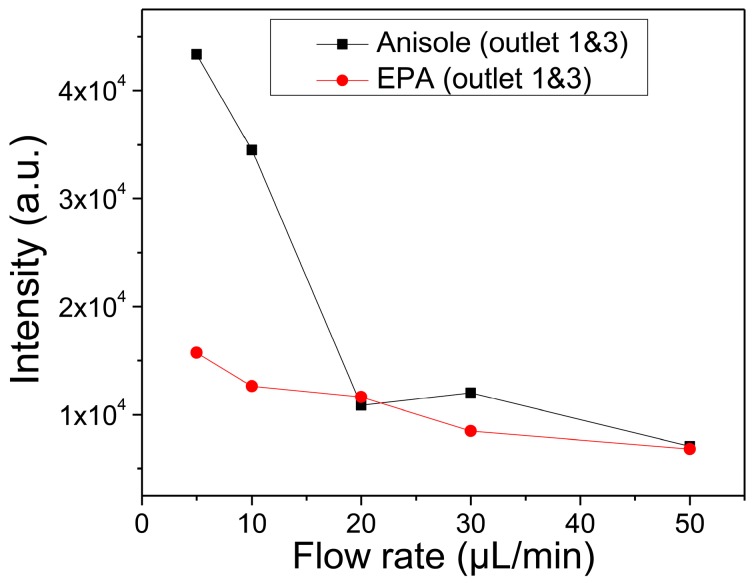
Anisole and EPA collected at the side outlets for different inlet flow rates.

Figure 6 presents qualitative separation efficiency of anisole and EPA in the outlet channels as a function of the inlet flow rate. Anisole diffused more rapidly from the focused flow to the side flow of the pure DMF solvent than EPA through the interface for the same inlet flow rates. As a result, the anisole must be collected with the higher amount at the side outlets. These explanations mean that the fractionation of anisole from the mixture with PS is more efficient than EPA. Additionally, it indicates that, when three of them are mixed, there is a possibility of separating anisole, EPA, and PS in turn.

The advantage of using the diffusion-based separation in the microchannel enables the continuous and flow-through separation. In this experimental evaluation, the possibility of separation in a simple configuration of the microchannel was challenged, and its possibility was shown successfully. Moreover, the efficiency can be improved simply by fabricating the successive separation channels.

In the experimental results, the separation might be coupled with the transverse migration of polymer chains by inertia in the shear flow [20]. To examine it near the flow interface of pure DMF and the DMF with polymers, the confinement length scale was checked. The hydrodynamic radius of PS in DMF was about 6.31 nm and the molecular diameter of anisole and EPA were in an Angstrom scale of ~0.1 nm. The height and width of the microchannel were 100 µm and 500 µm. Thus, they are in a weakly confined regime. In this regime, it is known that the equilibrium conformational statistics of the polymer chain are largely unchanged from bulk values.

**Figure 7:**
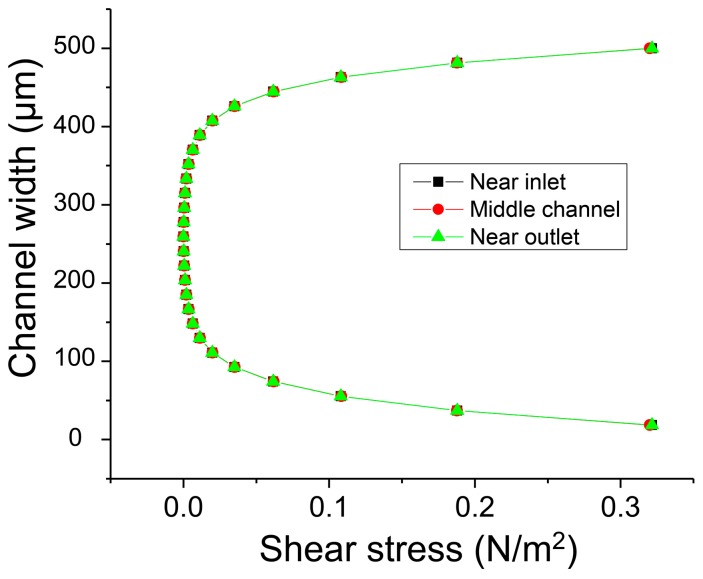
The distribution of shear stress along the microchannel.

Additionally, the distribution of shear stress in the microchannel was examined. The polymers dissolved in DMF were introduced through the central inlet channel to form the central laminar flow, and this flow was focused by the pure DMF flow injected from the side inlet channels. Thus, the width of the polymer solution was always controlled less than one third of the total channel width of 500 µm for the diffusion-based separation and was positioned in the center. Figure 7 shows clearly that the shear stress was almost zero in the range of ±150 µm from the channel center and that the polymer solution flows in the region of zero shear stress. Additionally, the distribution of the shear stress along the microchannel does not change because the flow is already fully developed. As a result, it is assumed that the shear stress negligibly influences the transverse diffusion of the molecules.

**Figure 8:**
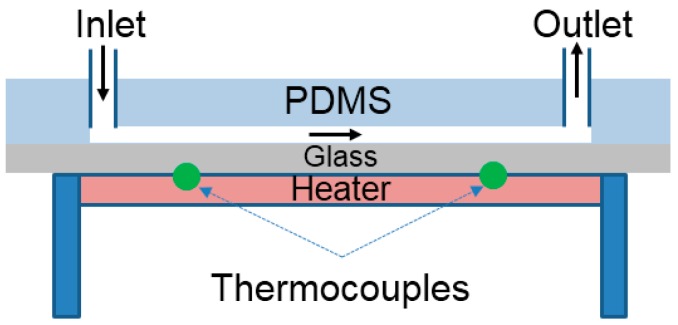
Experimental setup to evaluate the influence of temperature on separation.

In addition, the diffusion is influenced sensitively by temperature. When ambient temperature increases, the soluble substances diffuse in a livelier manner in a liquid solution. Thus, the transverse movement of PS and anisole dissolved in the solvent of DMF occurs fast through the interface of the lamination flow in the microfluidic channel. To evaluate the influence of temperature on the fractionation of anisole from the mixture, the microfluidic channel was set up in the temperature-controlled test rig as shown in Figure 8. The heater was embedded in the substrate, and two K-type thermocouples were installed near the inlet and outlet to measure the average ambient temperature. The temperature was controlled by the heater and the input power to the heater was regulated to obtain a steady and constant substrate temperature. It is well known that the microfluidic channel has the advantage of maintaining a temperature constant because of a short distance of conduction heat transfer [21,22].

The mixture of PS and anisole was used for the experiment. The substrate temperature was increased. The inlet flow rates of the mixture and the DMF solvent were 20 µL/min and 60 µL/min, respectively. Three different ambient temperatures of 22, 30, and 40 °C were examined. The separated samples collected from the outlets were analyzed using GPC.

**Figure 9:**
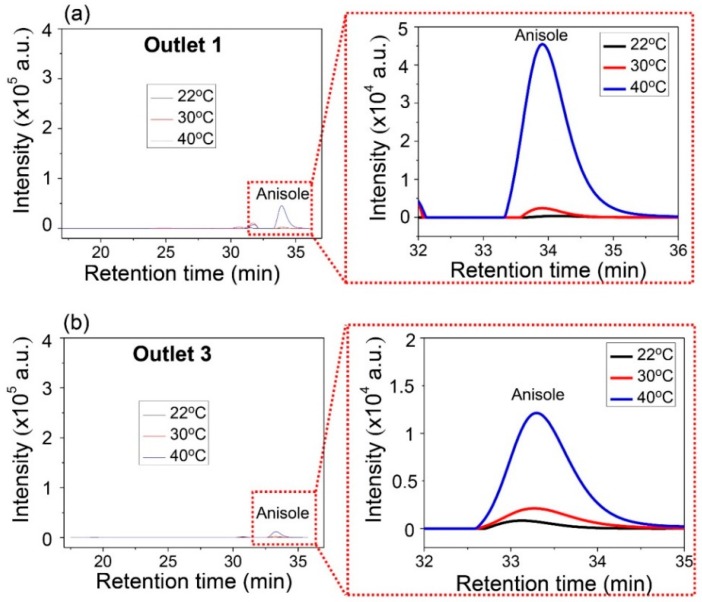
GPC results for the influence of temperature on separation at the same inlet flow rate of mixture and *N*,*N*-dimethylformamide (DMF) solvent: (**a**) GPC analysis of Outlet 1; (**b**) GPC analysis of Outlet 3.

Anisole was collected only from Outlets 1 and 3, and the concentration of anisole was examined for the different ambient temperatures. Figure 9 shows the comparative concentration of anisole. As the temperature increased, the high peak of intensity of anisole was measured at Outlets 1 and 3. This means that more anisole can be collected in higher temperature environments. Additionally, the peak signal of PS was not observed at Outlets 1 and 3. Under these experimental different temperatures, PS was heavy enough to stay in the central layer of the three-layered lamination flow in the microchannel. The results also indicate that the ambient temperature can be used to improve the separation efficiency of a light substance from the mixture with heavy substance.

According to the experimental results, the selective separation can be optimized by controlling the relaxation time of anisole and that of PS and their diffusivity. The relaxation time depends on the separation time associated with the flow rate and the diffusivity does on ambient temperature. The molecular weight of PS is about 425 times heavier than that of anisole and 280 times heavier than that of EPA. As a result, PS molecules are difficult to move through the interface of the two laminated solutions because of a larger relaxation time, which scale as ~M^1.76^ as described in Equation (1), even though a diffusivity is obtained at a higher temperature.

## 4. Conclusions

In this paper, we proposed a flow-through microfluidic method for the fractionation of small molecules in a mixture using different dynamic responses of mixture components to the microfluidic flow field. The numerical and experimental results of the PS and anisole mixture demonstrated that the fractionation of anisole from the mixture could be performed in a continuous way, which can be optimized by controlling the flow rate in the microfluidic channel and the ambient temperature. This method is advantageous in that the separation can be readily operated in a standard microfluidic device without extra external force, which indicates a high potential for commercialization. This method can be applied to separate monomers from aggregates.

## Abbreviations

The following abbreviations are used in this manuscript:

DMF	*N*,*N*-dimethylformamide
DNA	Deoxyribonucleic acid
EPA	Ethyl phenylacetate
Eq	Equation
GPC	Gel permeation chromatography
*M*_n_	Molecular weight
PDMS	Polydimethylsiloxane
PS	Polystyrene
